# Differential Gene Expression in Late-Onset Friedreich Ataxia: A Comparative Transcriptomic Analysis Between Symptomatic and Asymptomatic Sisters

**DOI:** 10.3390/ijms252111615

**Published:** 2024-10-29

**Authors:** Sara Petrillo, Alessia Perna, Andrea Quatrana, Gabriella Silvestri, Enrico Bertini, Fiorella Piemonte, Massimo Santoro

**Affiliations:** 1 Unit of Muscular and Neurodegenerative Diseases, Children’s Hospital Bambino Gesù, Scientific Institute for Research, Hospitalization and Healthcare (IRCCS), 00146 Rome, Italy; sara.petrillo@opbg.net (S.P.); a.quatrana@outlook.com (A.Q.); enricosilvio.bertini@opbg.net (E.B.); 2 Center for Neuromuscular and Neurological Rare Diseases, San Camillo Forlanini Hospital, 00152 Rome, Italy; alessia1perna@gmail.com; 3 Department of Neurosciences, Università Cattolica del Sacro Cuore, 00168 Rome, Italy; gabriella.silvestri@unicatt.it; 4 UOC of Neurology, Area of Neuroscience, Fondazione Policlinico Universitario A. Gemelli IRCCS, 00168 Rome, Italy; 5 Division of Biotechnologies, Italian National Agency for New Technologies, Energy and Sustainable Development (ENEA), 00123 Rome, Italy; massimo.santoro@enea.it

**Keywords:** Friedreich’s ataxia, RNA-seq, transcriptomic analysis, TLR4, inflammation, neurodegenerative disease, differentially expressed genes (DEGs)

## Abstract

Friedreich ataxia (FRDA) is the most common inherited ataxia, primarily impacting the nervous system and the heart. It is characterized by GAA repeat expansion in the FXN gene, leading to reduced mitochondrial frataxin levels. Previously, we described a family displaying two expanded GAA alleles, not only in the proband affected by late-onset FRDA but also in the younger asymptomatic sister. The molecular characterization of the expanded repeats showed that the affected sister carried two canonical uninterrupted GAA expended repeats, whereas the asymptomatic sister had a compound heterozygous for a canonical GAA repeat and an expanded GAAGGA motif. Therefore, we decided to perform RNA sequencing (RNA-seq) on fibroblasts from both sisters in order to understand whether some genes and/or pathways might be differently involved in the occurrence of FRDA clinical manifestation. The transcriptomic analysis revealed 398 differentially expressed genes. Notably, TLR4, IL20RB, and SLITRK5 were up-regulated, while TCF21 and GRIN2A were down-regulated, as validated by qRT-PCR. Gene ontology (GO) enrichment and network analysis highlighted significant involvement in immune response and neuronal functions. Our results, in particular, suggest that TLR4 may contribute to inflammation in FRDA, while IL20RB, SLITRK5, TCF21, and GRIN2A dysregulation may play roles in the disease pathogenesis. This study introduces new perspectives on the inflammatory and developmental aspects in FRDA, offering potential targets for therapeutic intervention.

## 1. Introduction

Friedreich ataxia (FRDA, OMIM #229300) is the most common inherited form of ataxia (1:50,000 individuals), caused by the abnormal expansion of the GAA repeat sequence in intron 1 of the FXN gene located on chromosome 9 (position 9q21.11), leading to reduced levels of frataxin, a mitochondrial protein involved in iron–sulfur cluster biosynthesis and crucial for mitochondrial energy production and protection against oxidative stress [1]. FRDA pathogenesis primarily affects the nervous system and the heart, causing progressive damage and resulting in an overall defect of iron trafficking and iron accumulation in mitochondria [2,3,4]. Until now, the exact function of the mitochondrial protein frataxin (FXN) in iron metabolism is still highly debated [1]. A role in the iron storage process was initially proposed, and many studies have highlighted a primary function for the protein in the Fe-S cluster biosynthesis, although several other FXN functions have been evidenced (iron chaperone, gate-keeper of detrimental Fe-S cluster biosynthesis, sulfide production stimulator, and sulfur transfer accelerator). The iron accumulation is not systematically observed and seems to be tissue-specific [5,6,7,8,9]. Thereby, no definitive conclusions on the exact function of FXN can be defined yet, even if a major function as “accelerator” for sulfur transfer in the Fe-S biosynthetic complex is now strongly emerging [1]. Oxidative stress in FRDA is most likely the result of a mitochondrial reactive oxygen species (ROS) overload generated by the iron-mediated Fenton reaction, and by an impaired signaling of the master antioxidant regulator nuclear factor erythroid 2-related factor 2 (NRF2) [10,11,12,13,14,15,16,17]. Importantly, a mitochondrial dysfunction, associated with the activation of type I interferon response, has been described in frataxin knockdown iPSC-derived cardiomyocytes and neurons [18,19], and in skeletal muscle of patients by transcriptome analysis [20,21,22,23], thus supporting a central role for mitochondria in the disease. This mitochondrial-driven redox imbalance leads to the activation of some iron-dependent oxidases (,i.e., ALOX15) in FRDA, catalyzing the oxidation of polyunsaturated fatty acids phospholipids (PUFA-PLs) and triggering ferroptosis [12,13,23,24,25].

In a previous report, we described a family displaying two small, expanded GAA alleles in the proband (female) affected by late-onset FRDA (LOFA), and in her asymptomatic younger sister [26]. We demonstrated that the asymptomatic sister had a compound heterozygote for an expanded GAA allele and an uncommon (GAAGGA) 66–67 repeat expansion; of note, expression studies showed that both sisters displayed a similar significant reduction in frataxin levels in leukocytes and fibroblasts, but only one sister developed clinical disease manifestation of FRDA. Moreover, both sisters exhibited slight differences in the size of the FXN alleles between leukocytes and fibroblasts, which indicates the presence of somatic mosaicism, suggesting that the FXN alleles underwent slight changes in size during tissue development or differentiation [26].

It is important to note that the GAA1 allele remained relatively stable not only across different tissues within individuals (indicating limited somatic variation) but also across generations, indicating a low rate of change during intergenerational transmission. This stability could potentially contribute to the asymptomatic presentation in the asymptomatic sister despite the presence of the expanded allele [26]. In a following study, we also explored whether the transcription factor NRF2, the first line of antioxidant defense in cells, might contribute to the pathological phenotype in the two sisters. We showed that NRF2 was highly activated in the asymptomatic sister, while it was constitutively low in the affected one, as expected in Patients with FRDA [16]. Therefore, our previous findings, together with those of several published papers, led us to hypothesize on the presence of other mechanisms that, despite the evidence of frataxin depletion, could contribute to the occurrence of neurological symptoms.

In this study, we tried to go deeper into the mechanisms differentiating the two sisters by performing RNA-seq transcriptomic analysis on fibroblasts obtained from both the symptomatic sister and the asymptomatic sister. The ultimate goal was to identify genes and/or pathways that are differently involved in the clinical manifestation of FRDA.

## 2. Results

### 2.1. Transcriptomic Analysis

**Figure 1:**
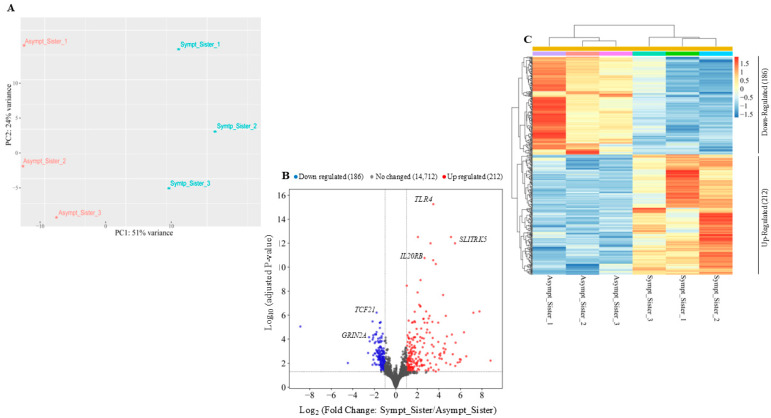
Identification of differentially expressed genes by comparing the symptomatic vs. the asymptomatic sister. (**A**) Principal component analysis (PCA) plotting for both sisters considering the top 500 most variable genes. (**B**) Volcano plot of the RNA-seq results using cutoff values of log2 fold change > ±1 and adjusted-*p*-value < 0.05. Non-changed genes are shown in gray, up-regulated genes are in red, and down-regulated genes are in blue. (**C**) Heatmap of differentially expressed genes. Hierarchical clustering was performed using the Pearson correlation distance metric and the average linkage clustering algorithm.

In order to identify genes differentially expressed between the symptomatic and the asymptomatic sister, RNA-seq was performed with three biological replicates from each individual, in collaboration with Genartis S.r.l. (www.genartis.it; accessed on 7 August 2024). Figure 1A shows a scatter plot representing the projections of the first two principal components (PC1 and PC2) derived from a principal component analysis (PCA). This analysis was performed on the expression data of the top 500 most variable genes. The scatter plot highlighted the differences in transcriptional profiling between the two sisters, as reflected in the distinct separation along the principal components (Figure 1A).

Using a log2 fold change cutoff of ±1 and an adjusted *p*-value < 0.05, we identified a total of 398 differentially expressed genes between the two sisters (Figure 1B). A heatmap of unsupervised hierarchical clustering, based on z-score of normalized expression values (RLOG) of differentially expressed genes (DEGs, adjusted-*p*-value < 0.05 and log2 fold change ± 1), revealed that 212 genes were up-regulated and 186 genes were down-regulated in the symptomatic sister compared to the asymptomatic sister (Figure 1C). Hierarchical clustering and RLOG were both generated with DESeq2.

**Figure 2:**
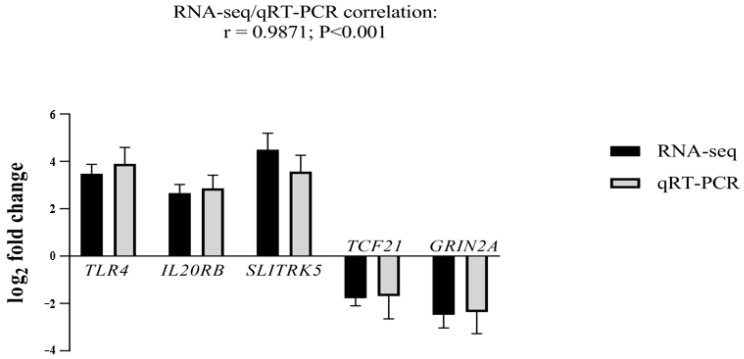
qRT-PCR analysis for validation of *TLR4*, *IL20RB*, *SLITRK5*, *TCF21,* and *GRIN2A* mRNA transcripts. Log_2_ fold changes determined by RNA-seq and qRT-PCR are presented as mean ± SD. Statistical significance was reached at *p* < 0.001 for all genes. Three independent fibroblast samples per sister were used (*n* = 3).

To validate our RNA-seq data, we selected the top five genes with the highest log2 fold changes and the highest significant adjusted *p*-value. Three genes were up-regulated: Toll-like receptor 4 (TLR4; log2 fold change = 3.5 and adjusted *p*-value = 6.71407 × 10^−20^), interleukin 20 receptor subunit beta (IL20RB; log2 fold change = 2.7 and adjusted *p*-value = 7.24718 × 10^−15^), SLIT and NTRK-Like Family Member 5 (SLITRK5; log2 fold change = 4.5 and adjusted *p*-value = 3.70368 × 10^−16^). Two genes were down-regulated: transcription factor 21 (TCF21; log2 fold change = −2.2 and adjusted *p*-value = 7.45383 × 10^−10^) and glutamate ionotropic receptor NMDA type subunit 2A (GRIN2A; log2 fold change = −2.5 and adjusted *p*-value = 2.78352 × 10^−7^) (Figure 2).

### 2.2. Gene Ontology (GO) and Pathway Analysis

**Figure 3:**
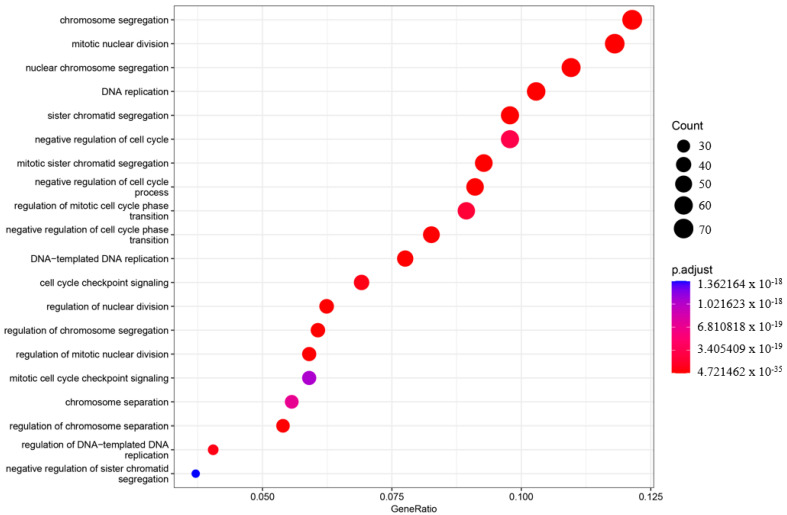
Gene ontology enrichment analysis (GO) of the biological process (BP). The top 20 enriched categories were plotted.

**Figure 4:**
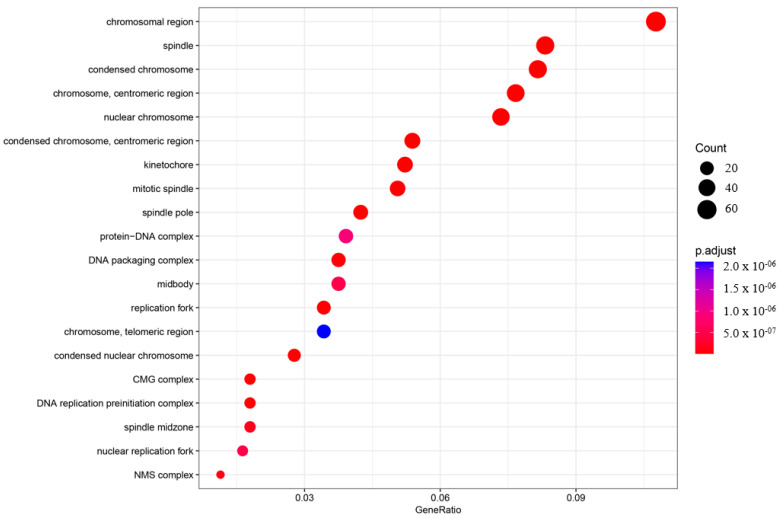
Gene ontology enrichment analysis (GO) of the cellular component (CC). The top 20 enriched categories were plotted.

**Figure 5:**
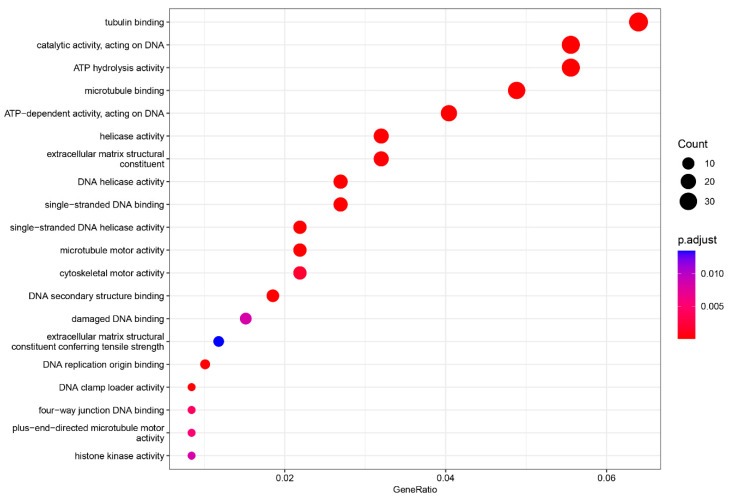
Gene ontology enrichment analysis (GO) of the molecular function (MF). The top 20 enriched categories were plotted.

In order to understand the roles of genes and their interactions in various biological processes when comparing the asymptomatic with the symptomatic sister, gene ontology (GO) enrichment analysis was performed using ClusterProfiler (v3.18.1) [27]. The enriched GO terms were categorized into three groups: biological process (BP), cellular component (CC), and molecular function (MF). The top 20 enriched categories for BP, CC, and MF were plotted (Figure 3, Figure 4 and Figure 5).

In the BP category, four terms showed very significant enrichment: chromosome segregation (adjusted *p*-value = 4.71997 × 10^−33^, count = 72), mitotic nuclear division (adjusted *p*-value = 4.72146 × 10^−35^, count = 70), nuclear chromosome segregation (adjusted *p*-value = 2.32768 × 10^−31^, count = 65), and DNA replication (adjusted *p*-value = 3.62835 × 10^−31^, count = 61) (Figure 3). The CC category also displayed significant pathway enrichment, particularly chromosomal region (adjusted *p*-value = 7.88197 × 10^−28^, count = 66) and condensed chromosome (adjusted *p*-value = 1.76047 × 10^−23^, count = 50) (Figure 4).

In the MF category, three terms were significantly enriched: tubulin binding (adjusted *p*-value = 5.88436 × 10^−11^, count = 38), catalytic activity, acting on DNA (adjusted *p*-value = 4.57436 × 10^−11^, count = 33), and ATP hydrolysis activity (adjusted *p*-value = 2.70806 × 10^−6^, count = 33) (Figure 5).

**Figure 6:**
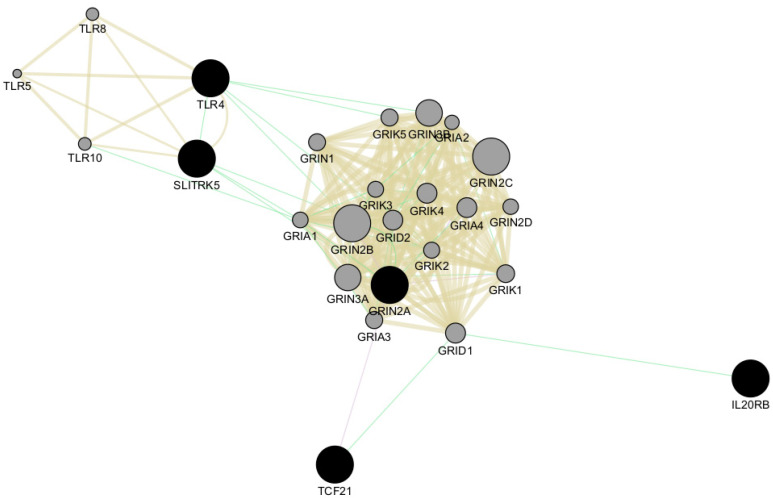
Network analysis of the top five genes using Cytoscape v3.10.1 software. The top five genes (*TLR4, SLITRK5, IL20RB*, *TCF1*, and *GRIN2A*) are represented as black circles. All genes involved in the network are indicated as grey circles. Light-brown lines indicate shared protein domains, green lines indicate co-expression, and violet lines indicate genetic interactions.

We also performed network analysis of the top five genes, TLR4, SLITRK5, IL20RB, TCF1, and GRIN2A, using Cytoscape v3.10.1 software [28]. This analysis revealed shared protein domains, primarily within the networks of glutamate receptor activity, transmitter-gated channel, and Toll-like receptor signaling pathways involving GRIN2A and TLR4, respectively (Figure 6). Additionally, Cytoscape analysis indicated co-expression of GRIN2A with TCF21 and genetic interactions among all five top genes (Figure 6).

## 3. Discussion

Several interesting papers have previously analyzed samples obtained from patients with FRDA by RNAseq. Among these, Indelicato et al. [20] evidenced a double hit in the skeletal muscle of patients, showing a profound mitochondrial failure, progressing over time. The skeletal muscle proteome analysis confirmed this mitochondrial signature, unveiling not only an extensive down-regulation of OXPHOS proteins, but also alterations in regulatory pathways [21,22]. A dysregulation of genes involved in interferon-induced apoptosis and in DNA damage has been also described in FRDA fibroblasts by RNA-Seq [29], and an inflammatory transcriptional signature was identified in peripheral blood of patients [30]. The transcriptomic analysis performed on sensory neuronal cultures from patients with FRDA [31,32] revealed cytoskeletal impairment of the growth cone, neurite extension, and, at later stages, defects of synaptic plasticity. Furthermore, the transcriptome analysis carrying out on iPSC-derived cardiomyocytes and neurons [18,19] confirmed the mitochondrial dysfunction and, importantly, evidenced the activation of type I interferon response, which was induced, at least in part, by the release of mitochondrial DNA into the cytosol, activating the cGAS-STING sensor pathway [18].

Thus, starting from these previous studies, we decided to compare the RNA-seq transcriptomic analyses of the two sisters, both of them displaying two expanded GAA alleles and low levels of frataxin, but only one showing the FRDA symptoms. In particular, the symptomatic sister was homozygous for two pathological GAA expansions of 206 (GAA1) and 473 (GAA2) repeats, while her asymptomatic sister was a compound heterozygous carrying two expanded alleles of 146 (GAA1) and 176 (GAA2) repeats and uncommon (GAAGGA)66–67 repeats [26]. The symptomatic sister was 43 years old, with symptoms starting at the age of 35. The asymptomatic one was 36 years old, and, during 4 years of follow-up, she did not develop any FRDA manifestation.

We identified 398 differentially expressed genes (212 up-regulated and 186 down-regulated) between the symptomatic patient and her asymptomatic sister. Among these, five genes exhibited the highest significant log2 fold changes and the most significant *p*-values were *TLR4, IL20RB*, *SLITRK5*, *TCF21*, and *GRIN2A*. In particular, *TLR4*, *IL20RB*, and *SLITRK5* were up-regulated, while *TCF21* and *GRIN2A* were down-regulated.

TLR4 (*603030, 9q33.1) plays a fundamental role in the innate immunity regulation by recognizing the pathogen-associated (PAMPs) and damage-associated (DAMPs) molecular patterns [33]. Its activation leads to two main signaling pathways: the MyD88-dependent and TRIF-dependent pathways. These are aimed at both defending against infections and responding to cellular damage [34]. In addition, TLR4 displays an important role in the central nervous system by mediating the activation of the inflammatory nuclear factor-κB (NF-κB), as documented in several neurodegenerative diseases such as Alzheimer’s, Parkinson’s, Huntington’s diseases, and Amyotrophic Lateral Sclerosis [35].

NF-kB is sensitive to redox-status changes and its immune function is modulated by oxidative stress [36,37]. Oxidative stress, associated with a deficiency of the antioxidant factor NRF2, underlies the pathogenic mechanism in FRDA [16], and NRF2, besides its essential role as main regulator of redox homeostasis, is also a fine modulator of the inflammatory response, by interacting with the TLR4/NF-kB pathway. Indeed, NRF2 is able to restrain the TLR4-mediated inflammation through the induction of antioxidant proteins and/or the inhibition of pro-inflammatory cytokines [38], and by its direct functional cross-talk with NF-κB [39]. Therefore, the up-regulation of the TLR4 gene that we found in the symptomatic sister may be indicative of a redox-mediated neuro-inflammatory response in FRDA, with TLR4 as a trigger itself. Further experiments will be needed to evaluate whether this increase in TLR4 expression is sufficient for its activation.

Oxidative stress is the primary pathological mechanism of neuronal death in FRDA; nevertheless, neuro-inflammation has been proposed to accompany, and even contribute to, neuropathology in FRDA [30]. Signs of neuro-inflammation have been reported in patients’ samples, and in cellular and animal models of FRDA [30,40,41], and some studies have in vivo quantified brain neuro-inflammation, alongside peripheral plasma cytokine measures [41]. Interestingly, it is emerging that, although the pathological manifestations in FRDA are primarily observed in neurons, the surrounding non-neuronal cells may give an important contribution to the pathogenesis of the disease, by secondary gliotic events triggered by the iron accumulation [32]. Indeed, the accumulation of iron in FRDA leads to a defensive glial reaction finalized to protect neurons from oxidation, but the glial cells themselves may amplify the neuronal damage and contribute to neuronal death by further increasing iron accumulation in the nervous system. The glia acts as scavenger of iron overload but, in case of iron dysmetabolism, it may overproduce ROS, exacerbating the oxidative-mediated inflammation. Moreover, ferroptosis, which underlies pathogenesis in FRDA, has been demonstrated to play a role in regulating the inflammatory response in several neuro-immune and neurodegenerative diseases [42].

In the cerebellar tissues of patients, marked astrogliosis of the dentate nucleus has been reported, as demonstrated by ferritin positive astrocytes detected near the vessel walls [43]. Moreover, the autopsy specimens of patients with FRDA showed the intrusion of CNS-derived astroglia into the dorsal roots [44] and plasma levels of glial fibrillary acidic protein (GFAP) are significantly higher in patients with FRDA, thus reflecting a consistent glial activation [45]. The cerebellar astrocytes are highly susceptible to the FXN deficiency [46], displaying abnormal secretion of IL-6 and macrophage inflammatory protein-1 alpha (MIP-1α). Also, oligodendroglia and Schwann cells are vulnerable to FXN knockdown [43], as demonstrated by microarrays analysis showing a decrease in antioxidant genes and a consistent increase in inflammatory cytokines (IL-1β, IL-1α, IL-6, NFκB, TNF) that may contribute to DRG neuron loss [47].

Overall, the evidence supports a strong involvement of the neuro-inflammatory mechanism in FRDA; our results, comparing a symptomatic sister with her asymptomatic sister, may help to highlight the importance of inflammation in the development of clinical symptoms, although the role of neuro-inflammation, as a cause or consequence of the disease onset and progression, still needs to be defined. Nevertheless, investigating the role of neuro-inflammation in FRDA is crucial for the comprehension of the disease onset and progression, and the modulation of inflammation might represent one of the most promising therapeutic strategies.

Together with *TLR4*, four additional genes emerged by RNA-Seq as dysregulated in the symptomatic patient with respect to the asymptomatic one. *IL-20RB* and *SLITRK5* were found to be up-regulated, while *TCF21* and *GRIN2A* were down-regulated. We explored, by reviewing the literature, the function of these abnormally expressed genes, to try to better understand their role in the disease.

IL-20 is a cytokine belonging to the IL-10 family and its receptor IL-20RB was found to be significantly up-regulated in inflammatory diseases, such as psoriasis and rheumatoid arthritis, indicating its involvement in promoting innate immune responses [48]. The IL20R signaling modulates the transcription factor STAT3, thus eliciting an anti-inflammatory activity [49], but it also plays a role in the type I IFN/STAT2-mediated death during inflammation [50]; interestingly, the type-I IFN response is known to be redox-modulated by NRF2 [51]. By RNASeq, we found a consistent increase in IL-20RB expression in the symptomatic sister compared with the unaffected one, thus supporting our hypothesis of an inflammatory response activation in FRDA. To the best of our knowledge, so far, no research study has described any direct interaction between TLR4 and IL-20RB, but we cannot rule out the idea that both signaling pathways might be implicated simultaneously in the disease, contributing to the exacerbation of the disease’s activity.

From the comparison between the affected and the unaffected sister, other three differently modulated genes were identified by RNASeq: SLITRK5 (up-regulated), GRIN2A, and TCF21 (both down-regulated).

SLITRK5, one of the six members of the SliTrk protein family, is widely expressed in the CNS, regulating many essential steps of CNS development, such as axon and dendritic growth, neuron differentiation, and synaptogenesis. It has been variously involved in the pathogenesis of several neurological diseases, including the obsessive compulsive disorder (OCD), attention deficit/hyperactivity disorder (ADHD), glioma, autism spectrum disorders (ASDs), Parkinson’s disease (PD), and drug-refractory temporal lobe epilepsy (TLE), pointing to SLITRK5 being a potential new target for the treatment of several CNS diseases [52,53].

Similarly to *SLITRK5*, *GRIN2A* is another gene involved in some neurological functions; GRIN2A is a component of N-methyl-D-aspartate receptor (NMDAR) that mediates the fast-excitatory neurotransmission in CNS and is involved in maturation and phenotypic maintenance of parvalbumin interneurons (PVIs) [54,55]. Defects in GRIN2A are related to many neurological disorders, including developmental delay, evolving to intellectual disability (DD/ID), epilepsy, speech and language disorders, movement disorders, and neuropsychiatric disorders [56]. Interestingly, GRIN2A KO mice display high susceptibility to redox dysregulation and alterations in antioxidant systems in the prefrontal cortex, especially when an oxidative insult was applied during early postnatal development [54]. Authors demonstrated that these effects were long-lasting but preventable by the antioxidant N-acetylcysteine.

Another gene found to be down-regulated in the symptomatic sister with FRDA is the *transcription factor 21* (*TCF21*). It mediates cell fate and differentiation during organogenesis, playing a crucial role in the epithelial–mesenchymal transition (EMT), cell cycle, autophagy, proliferation, differentiation, and cell survival [57]. Importantly, TCF21 plays a critical role in cardiovascular diseases by regulating the differentiation of epicardium [58], and its absence may cause morphological epicardial defects in the early stage of cardiac development [59]. Overall, and based also on such data from the literature, the dysregulated expression of *SLITRK5, GRIN2A*, and *TCF21*, documented in fibroblasts of the symptomatic patient compared with her asymptomatic sister, highlight a role for these genes in the neurological (*SLITRK5* and *GRIN2A*) and cardiac (*TCF21*) pathology occurring in FRDA.

In conclusion, our study is the first to evidence an abnormal expression of inflammatory (*IL-20R* and *TLR4*) and developmental genes (*SLITRK5*, *GRIN2A*, and *TCF21*) in FRDA. If confirmed in further studies, these findings may contribute to the discovery of new disease targets, which may potentially be useful in developing combined therapies in FRDA.

## 4. Materials and Methods

### 4.1. Subjects

This study was conducted in accordance with the Declaration of Helsinki, and its design fulfilled the guidelines of all involved institutional ethical boards. RNA samples were extracted from cultured fibroblasts isolated from punch skin biopsies, previously obtained after written informed consent, from two sisters belonging to am FRDA family described in detail previously [16,26]. Briefly, the family proband (female) was a 43-year-old person who had a diagnosis of LOFA, manifested as a slowly progressive spastic ataxia starting from the age of 35, who resulted homozygous for two pathological expansions in the GAA repeat region [26]. Her asymptomatic 36-year-old sister was a compound heterozygous carrying a common expanded GAA allele and an uncommon (GAAGGA)66–67 repeat; during 4 years of follow-up, she did not develop any FRDA manifestation [26].

### 4.2. Fibroblasts Cultures

Skin biopsies were isolated from the proband, who was a clinically and genetically proven FRDA patient, and from her asymptomatic sister. Fibroblasts were grown in Dulbecco’s modified Eagle’s medium supplemented with 10% fetal bovine serum, 50 units/mL penicillin, 50 µg/mL streptomycin, and 0.4% (*v*/*v*) amphotericin B (250 µg/mL), at 37 °C in 5% CO_2_. Cells at passages 9–11 were used and tested for mycoplasma contamination using Venor GeM qEP kit (MB Minerva Biolabs, Berlin, Germany), with negative results.

### 4.3. RNA-Seq Analysis

Total RNA was extracted from three independent fibroblast samples per sister using the Total RNA Purification Plus Kit (Norgen Biotek, Thorold, ON, Canada), according to the manufacturer’s protocol. RNA quantification was performed on a NanoDrop2000 Spectrophotometer (Thermo Scientific, Waltham, MA, USA). The purity of RNA was assessed by measuring the 260/280 nm absorbance ratio. RNA integrity was assessed using the RNA 6000 Nano Kit on a Bioanalyzer (Agilent Technologies, Santa Clara, CA, USA). The RNA integrity number (RIN) was higher than 9 for all samples.

Illumina RNA-seq libraries were generated using the TruSeq stranded mRNA ligation kit (Illumina, San Diego, CA, USA) from RNA samples, after poly(A) capture and according to manufacturer’s instructions. The starting input of RNA samples was 750 ng. Quality and size of RNA-seq libraries were assessed by capillary electrophoretic analysis with the Agilent 4150 Tape station (Agilent Technologies, Santa Clara, CA, USA). Libraries were quantified by real-time PCR against a standard curve with the KAPA Library Quantification Kit (KapaBiosystems, Wilmington, MA, USA). Illumina Sequencing Libraries were pooled at equimolar concentration and sequenced with Illumina technology generating on average 29.8 million fragments in 150-bp paired-end mode on a Novaseq 6000 sequencer (Illumina, San Diego, CA, USA). All experiments were performed in triplicate.

### 4.4. Bioinformatic Analysis

The quality of the reads was assessed using FastQC v 0.12.0 software (http://www.bioinformatics.babraham.ac.uk/projects/fastqc/, accessed on 7 August 2024). Raw reads were trimmed with fastp (v.0.21.0) [60] with trim_poly_x parameter, to remove adapters and low-quality bases with default parameters. Filtered reads were aligned to the Homo sapiens reference genome hg38 using STAR aligner (v2.7.9a) [61] with parameter peOverlapNbasesMin 5. Reads distribution was conducted on CDS, and intronic and intergenic were computed using reads_distribution.py script from RSeQC suite (v4.0.0) [62]. Gene expression quantification has been performed using RSEM (v1.3.1) (https://github.com/deweylab/RSEM, accessed on 7 August 2024) and the Homo sapiens Ensembl v109 annotation. Genes level abundance estimated counts and gene length obtained with RSEM were summarized into a matrix using the R package tximport (within DESeq2 package); subsequently, differential expression analysis was performed with DESeq2 (1.30.1) [63]. To generate more accurate Log2 Fold Change estimates for low-expressed genes, the shrinkage of the Log2 Fold Change was performed applying the apeglm method [64]. Finally, a Gene Ontology (GO) enrichment analysis of DEGs with adjusted *p*-value < 0.05 was performed using ClusterProfiler (v3.18.1) [27]. The data analysis was visualized using a volcano plot generated on the SRplot platform [65]. This plot combined the Log10 adjusted *p*-values (y-axis) with the Log2 fold changes (x-axis) to identify genes that exhibited statistically significant changes in expression levels.

Finally, network analysis was performed with Cytoscape v3.10.1 software [28]. Specifically, the GeneMANIA Cytoscape app was used to construct a composite gene–gene functional interaction network from genes identified through transcriptomic analysis.

### 4.5. Quantitative Real-Time PCR (qRT-PCR) Validation

qRT-PCR was used to validate the expression changes observed for the top five genes when comparing symptomatic versus asymptomatic sisters. Total RNA was extracted from three independent fibroblast samples for each sister and (1000 ng) was reverse transcribed with the SuperScript First-Strand Synthesis system with random hexamers as primers (Thermo Fisher Scientific, Waltham, MA, USA). According to MIQE guidelines [66], qRT-PCR was performed using TaqMan Universal Master Mix II (Thermo Fisher Scientific, Waltham, MA, USA) and TaqMan probe assays were performed for *TLR4* (assay number Hs00152939_m1, Thermo Fisher Scientific, Waltham, MA, USA), *IL20RB* (assay number Hs00376373_m1, Thermo Fisher Scientific, Waltham, MA, USA), *SLITRK5* (assay number Hs01007362_s1, Thermo Fisher Scientific, Waltham, MA, USA), *TCF21* (assay number Hs00162646_m1, Thermo Fisher Scientific, Waltham, MA, USA), and *GRIN2A* (assay number Hs00168219_m1, Thermo Fisher Scientific, Waltham, MA, USA). Gene expression levels were measured by qRT-PCR in an ABI PRISM 7500 Sequence Detection System (Thermo Fisher Scientific, Waltham, MA, USA). Data were analyzed using the 2^−∆∆Ct^ method with TBP (assay number Hs00427620_m1, Thermo Fisher Scientific, Waltham, MA, USA) and GAPDH (assay number Hs02786624_g1, Thermo Fisher Scientific, Waltham, MA, USA) as housekeeping genes, and shown as fold change relative to the asymptomatic sister. All PCR reactions were performed in triplicate and data were expressed as the mean ± standard error (SE).

### 4.6. Statistical Analysis

Statistical analysis was performed using GRAPHPAD/Prism 5.0 Software (GraphPad Company, San Diego, CA, USA). Statistically significant differences between the asymptomatic and symptomatic sisters were analyzed using Student’s *t*-test for normally distributed variables. All data are presented as mean ± standard error (SE). Statistical significance was defined as * *p* < 0.05, ** *p* < 0.01, and *** *p* < 0.001.

## Data Availability

Data are unavailable due to privacy or ethical restrictions.
